# Food consumption by NOVA food classification, metabolic outcomes, and barriers to healthy food consumption among university students

**DOI:** 10.1002/fsn3.3894

**Published:** 2023-12-10

**Authors:** Elizabeth Sekyi, Nana Ama Frimpomaa Agyapong, Guy Eshun

**Affiliations:** ^1^ Department of Hospitality Management Takoradi Technical University Takoradi Ghana; ^2^ Department of Food and Nutrition Education, Faculty of Home Economics Education University of Education Winneba Ghana; ^3^ Department of Clinical Nutrition and Dietetics, College of Health and Allied Sciences University of Cape Coast Cape Coast Ghana

**Keywords:** blood pressure, body mass index, NOVA foods, students, waist circumference

## Abstract

The NOVA food classification system is a simple tool that can be used to assess the consumption levels of different categories of foods based on their level of processing. The degree to which food is processed has a significant impact on health outcomes. In Ghana, no study exists on the consumption of the different NOVA food groups among tertiary students and how it relates to their metabolic outcomes. This study assessed the frequency of food intake according to the NOVA classification and how they relate to body mass index, waist circumference, and blood pressure. The barriers to the consumption of healthy foods among students were also assessed. This was a cross‐sectional study conducted among 352 students of the Takoradi Technical University. Questionnaire was used to obtain sociodemographic information as well as data on perceived barriers to healthy food consumption. Food frequency questionnaire was used to obtain data on dietary intake. The weight, height, waist circumference, waist‐to‐hip ratio, and blood pressure of all participants were measured. Chi‐square was used to compare categorical variables between males and females and to determine the association between the frequency of food intake according to the NOVA classification and metabolic indicators. The prevalence of overweight and obesity was 23.8%. More than half (51.1%) of the students had elevated blood pressure. The majority of study participants (54.2%) had a high frequency of consumption of both unprocessed and ultra‐processed foods. Male students who frequently consumed ultra‐processed foods (1–6 times/day) had significantly high blood pressure. High consumption of both ultra‐processed and unprocessed foods was also associated with elevated blood pressure among male students. Limited time to prepare healthy meals and the high cost of unprocessed foods were among barriers to which most students strongly agreed to. Establishment of canteens that provide affordable healthy foods, teaching students time management, and nutrition education can mitigate barriers to healthy food consumption.

## Graphical abstract

The majority of study participants had a high frequency of consumption of both unprocessed and ultra‐processed foods. High consumption of both unprocessed and ultra‐processed foods was also associated with blood pressure.

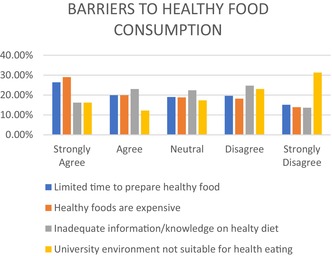

## 1 INTRODUCTION

Dietary guidelines based on food groups and nutrient profiles provide nutritional information and recommendations aimed at assisting the global population to make healthy food choices (FAO, 2016). These recommendations are geared toward the prevention of deficiency diseases, obesity, and other diet‐related noncommunicable diseases (Menegassi et al., 2019). Classifying foods based on the nutrients they contain can be misleading considering that the level of processing of various foods can also impact a person's health. Chronic non‐communicable diseases are currently the world's leading cause of morbidity and mortality and account for about 17.9 million deaths each year (Roth et al., 2018). They are mainly attributed to unhealthy diets, especially those that are ultra‐processed (Chen et al., 2020; Pagliai et al., 2021).

The NOVA food classification system was developed by the Centre for Epidemiological Studies in Health and Nutrition, School of Public Health, University of Sao Paulo, Brazil (Monteiro et al., 2018). This classification system groups foods based on their level of processing rather than by their nutrient profile. The NOVA food classification is based on the physical, biological, and chemical processes that foods are taken through after they are separated from nature and before they are consumed or used in the preparation of dishes and meals. There are four classes within the NOVA food system: unprocessed or minimally processed foods, culinary ingredients, processed foods, and ultra‐processed foods. Unprocessed foods comprise those that have undergone cleaning and removal of inedible or unwanted parts without the addition of oils, fats, sugar, and salt. Culinary ingredients include oils, butter, sugar, and salt. Processed foods have been preserved or improved by adding culinary ingredients. The fourth category includes industrial formulations made mostly or entirely from substances derived from or extracted from food constituents or synthesized in laboratories from food substrates or other organic sources. Normally, natural foods make up a small percentage of ultra‐processed products or are entirely absent.

Adolescents tend to increase their consumption of ultra‐processed foods and beverages when they enter university and other tertiary institutions. This increase in the consumption of ultra‐processed foods and beverages is often a result of independence from their families, convenience, and inadequate time to cook owing to the demands of academic work. The aforementioned factors culminate in opting for fast foods, and low‐nutrient dense cuisines, which puts them at risk of adverse health outcomes (Sprake et al., 2018). The adoption of such a dietary pattern is of public health concern since it has a long‐term impact on their health and well‐being. Being overweight or obese during adolescence and young adulthood is also a strong predictor of obesity and associated chronic diseases later in life (Abdelhafez et al., 2020; Isa & Masuri, 2011; Momanyi & Akinyi, 2014).

Currently, in Ghana, there are no data or research on the level of consumption of the different NOVA food groups among university students and how they are associated with health outcomes such as blood pressure and obesity. There is also a dearth of information with regard to the barriers these university students face in the acquisition of healthy foods within the university campus.

Therefore, this study sought to assess the frequency of food intake according to the NOVA classification and their association with overweight, obesity, and blood pressure as well as barriers to healthy food consumption among students of the Takoradi Technical University in Ghana.

## 2 METHODS

This was a cross‐sectional study that was undertaken among students of the Takoradi Technical University in Ghana. Trained research assistants assisted in the collection of data. Questionnaires were used to collect information on sociodemographics, meal consumption patterns, and barriers to the consumption of healthy foods. The questionnaire was pretested among nursing and midwifery students. Feedback from pretesting was used to modify the questionnaire prior to actual data collection. Weight, height, waist circumference, and body composition measurements of all participants were taken. Blood pressure measurements were also taken for all participants.

### 2.1 Study area

The study was conducted in the Takoradi Technical University which is within the Western Region of Ghana. Data collection was done within the various Faculties of the university.

### 2.2 Sample and sampling technique

Krejcie and Morgan sample size estimation formula was used to estimate the sample size for the study. The total population of students at the Takoradi Technical University was obtained from the students' records as 15,106. The total student population of each Faculty was also obtained. The sample size determined from the Krejcie and Morgan sample size table for the given population was between 306 and 310 (Chuan & Penyelidikan, 2006). Two percent of each faculty's population was calculated and all added to form the total sample of 302. The actual number of students who partook in this study was 352. Data collection took place within the faculties and within each faculty, study participants were randomly selected.

### 2.3 NOVA food consumption assessment

A detailed food frequency questionnaire was used to collect data on food consumption. The Prospective Urban Rural Epidemiology (PURE) study food frequency questionnaire which is validated was adapted for the study (Baker et al., 2020). The food item list consisted of unprocessed and processed foods. The NOVA food classification system was used to categorize foods as unprocessed, processed,culinary ingredients and ultra‐processed foods. The foods included in the food frequency questionnaire were typical local and exotic foods consumed in Ghana. The foods were grouped under the four NOVA food classes. For each food item, participants were to indicate the frequency at which they consumed it, whether daily (1–3 times daily), weekly (1–3 times per week), monthly (1–2 times per month), occasionally (once in several months), or never. These five responses were coded from 1 to 5. To assess the average frequency of consumption of each NOVA class, the response codes for foods within the same NOVA category were summed up and divided by the total number of foods within the category. The average responses were grouped into four; daily, weekly, monthly, and occasionally/never. These four responses were subsequently categorized into two for the unprocessed and ultraprocessed foods categories. Daily to weekly consumption were categorized as high frequency of consumption and coded as 1 while monthly and occasionally/never were categorized as low frequency of consumption and coded as 2. The codes for the ultra‐processed and unprocessed foods were subsequently computed by addition. A resulting code of 2 was interpreted as high frequency of consumption of both ultra‐processed foods and unprocessed foods, 3 as low and high consumption of either and 4 as low frequency of consumption of both.

### 2.4 Anthropometric measurement

Weight and height of all participants were measured following standard procedures. All participants were made to empty their pockets and remove cardigans and other heavy clothing. The Salter digital weight scale, model 9102 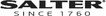, was used to measure the weight of all study participants. The scale was put on a hard leveled flat surface and set to 0. Participants were made to stand motionless on the weight scale with arms by the side and weight was measured to the nearest 0.1 kg. The Seca 213 portable stadiometer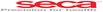 was used to assess the height of all study participants. Participants were made to stand upright on the stadiometer with their buttocks and upper back against the rod of the stadiometer, and heels against the base plate. Participants were also asked to look straight ahead to achieve a Frankfort plane, with shoulders relaxed and arms by the side. The head plate of the stadiometre was then pulled down to touch the highest point of the skull and height was taken to the nearest 0.1 cm. The height and weight of participants were used to calculate their Body Mass Index (BMI) by dividing their weight in kilograms by their height in meters squared (m^2^). Participants were subsequently categorized as underweight (<18.5 kg/m^2^), normal (18.5–24.9 kg/m^2^), overweight (25–29.9 kg/m^2^), and obese (≥30 kg/m^2^) as per the World Health Organization's standard (World Health Organization, 2010).

### 2.5 Blood pressure measurement

Blood pressure measurements were taken following standardized procedures. The blood pressure measurement of all participants was taken with the Omron M7 Intellisense digital sphygmomanometer 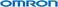. Participants were made to sit and rest for about 5 minutes after they had gone through the anthropometric measurements. Blood pressure was taken once and recorded for all participants. Blood pressure below 120 and 80 mm Hg for systolic and diastolic was classified as normal. Blood pressure measurement ≥120 mm Hg systolic and/or ≥80 mm Hg diastolic was classified as elevated blood pressure (World Health Organization, 2023).

### 2.6 Barriers to healthy food consumption

Perceived barriers to the consumption of healthy foods were assessed using a Likert scale questionnaire. The items in the questionnaire were based on common barriers to healthy food consumption reported among adolescents and young adults, specifically in Africa (Abdelhafez et al., 2020; Lima et al., 2021; Musaiger et al., 2013). Students were to indicate their level of agreement with a set of barriers provided within the questionnaire. The responses ranged from strongly agree to strongly disagree. Additionally, participants were also asked about the period within the day they had adequate time to eat, what constituted their favorite meals, if they planned their meals in advance, and if they consumed breakfast before leaving their hostels for lectures.

### 2.7 Ethical clearance

Ethical clearance for the study was granted by the Cape Coast Teaching Hospital Institutional Review Board (CCTHERC/EC/2022/106) in Ghana. Written permission was sought and obtained from the university authorities prior to data collection. Participants were made to sign an informed consent form to indicate voluntary participation.

### 2.8 Statistical analysis

Statistical Package for Social Sciences (IBM SPSS) version 23 was used for data analysis. Pearson's chi‐square and Fisher's exact tests were used to compare categorical variables between males and females. Independent sample *t*‐test was used to determine the differences among males and females for continuous variables such as weight and height. A *p*‐value of <.05 was used for statistical significance. Binary logistic regression was used to model the factors associated with blood pressure. Variables included in the model were age, gender, body mass index, and waist‐to‐hip ratio. The consumption levels of unprocessed foods and ultra‐processed foods were also included in the model.

## 3 RESULTS

A total of 352 students participated in the study. The mean age of study participants was 22.3 ± 2.4 years and the majority resided in hostels (49.7%). The majority of participants were single (99.1%) and Christians (92.6%). For the anthropometric characteristics, the mean weight and BMI of participants were 64.3 ± 10.6 kg and 21.8 ± 3.4 kg/m^2^, respectively. About 10% of study participants were underweight, 65.9% were of normal weight, and about a quarter (23.8%) were overweight or obese. The prevalence of overweight and obesity was significantly higher in females (37.2%) compared to males (13.6%; *p* > .001). The mean systolic and diastolic blood pressure were 122.8 ± 13.9 and 71.2 ± 10.4 mm Hg and systolic blood pressure was significantly higher among males (122.8 ± 13.9 mm Hg) compared to females (115.0 ± 11.4 mm Hg; *p* < .001). Table 1 shows the descriptive characteristics including sociodemographic characteristics and anthropometric measures of study participants.

**TABLE 1:** Descriptive statistics.

Variables	Total *N* = 352	Statistics and balance check		
	*N* (%) mean ± SD	Male *n* = 199 (56.5%) *n* (%)/mean ± SD	Female *n* = 153 (43.5%), *n* (%)/mean ± SD	*p*‐value
Age	22.3 ± 2.4	22.4 ± 2.5	22.2 ± 2.3	.513
Academic year				
1	100 (28.4)	55 (27.6)	44 (28.8)	**.025**
2	91 (25.9)	62 (31.2)	29 (19.0)	
3	161 (45.7)	81 (40.7)	80 (52.3)	
Faculty				
Applied Sciences	103 (29.3)	39 (19.6)	64 (41.8)	**<.001**
Engineering	93 (26.4)	82 (41.2)	11 (7.2)	
Applied Arts and Technology	49 (13.9)	27 (13,6)	22 (14.4)	
Built and Natural Environment	19 (5.4)	9 (4.5)	10 (6.5)	
Business Studies	88 (25.0)	42 (21.1)	46 (30.1)	
Marital status				
Single	349 (99.1)	198 (99.5)	151 (98.7)	.582
Married	3 (0.9)	1 (0.5)	2 (1.3)	
Religion				
Christian	326 (92.6)	183 (92.0)	143 (93.5)	.838
Muslim	24 (6.8)	15 (7.5)	9 (5.9)	
Others	2 (0.6)	1 (0.5)	1 (0.7)	
Residential status				
On Campus	79 (22.4)	44 (22.1)	35 (22.9)	.493
Off‐Campus (Hostel)	175 (49.7)	104 (52.3)	71 (46.4)	
Home	98 (27.8)	51 (25.6)	47 (30.7)	
Weight category				
Underweight	36 (10.2)	14 (7.0)	22 (14.4)	**<.001**
Normal	232 (65.9)	158 (79.4))	74 (48.4)	
Overweight	60 (17.0)	22 (11.1)	38 (24.8)	
Obese	24 (6.8)	5 (2.5)	19 (12.4)	
Waist circumference category				
Normal	325 (92.6)	193 (97.0)	132 (86.8)	**<.001**
Obese	26 (7.40)	6 (3.0)	20 s(13.2)	
Blood pressure category				
Normal (<120/80 mm Hg)	172 (48.9)	78 (39.2)	94 (61.4)	**<.001**
Elevated (>/=120/80 mm Hg)	190 (51.1)	121 (60.8)	59 (38.6)	
Monthly allowance in Ghana cedis	356.6 ± 251.3	374.4 ± 288.9	331.6 ± 184.4	.131
Weight (kg)	63.7 ± 12.0	64.3 ± 10.6	62.8 ± 13.6	.253
Height (cm)	167.3 ± 12.0	171.8 ± 7.1	161.3 ± 14.3	**<.001**
BMI (kg/m^2^)	22.6 ± 4.2	21.8 ± 3.4	23.7 ± 4.9	**<.001**
Waist circumference (cm)	76.3 ± 10.6	75.9 ± 11.4	76.9 ± 9.5	.366
Hip circumference (cm)	95.9 ± 9.8	94.1 ± 8.3	98.4 ± 11.0	**<.001**
Waist‐to‐hip ratio	1.4 ± 6.9	0.8 ± 0.07	2.3 ± 10.5	.050
Systolic blood pressure (mm/Hg)	119.4 ± 13.5	122.8 ± 13.9	115.0 ± 11.4	**<.001**
Diastolic blood pressure (mm/Hg)	71.0 ± 10	71.2 ± 10.4	70.5 ± 9.5	.482

*Note*: Bold values indicate p values ≤ 0.05.

Table 2 shows the consumption pattern of the NOVA food categories by gender. For unprocessed foods, most participants consumed them 1–6 times per day (41.2%) and 1–3 times per week (38.9%). This pattern of consumption was similar for culinary ingredients. For ultra‐processed foods, most students consumed it monthly (31.8%) and 1–6 times daily (29.5%). The consumption frequency of ultra‐processed foods was significantly higher among females compared to males.

**TABLE 2:** Consumption pattern of NOVA food category by gender.

NOVA categories and frequency of consumption	Total, *N* (%) *N* = 352 (100)	Males, *n* (%)	Females, *n* (%)	*p*‐value
Unprocessed/Minimally processed				
1–6 times/day	145 (41.2)	77 (38.7)	68 (45.0)	.527
1–3 times/week	137 (38.9)	83 (41.7)	54 (35.8)	
1–3 times/month	61 (17.3)	36 (18.1)	25 (16.6)	
Occasionally/never	7 (2.0)	3 (1.5)	4 (2.6)	
Culinary ingredients				
1–6 times/day	236 (67.0)	121 (60.8)	115 (75.7)	**.003**
1–3 times/week	71 (20.2)	43 (21.6)	28 (18.4)	
1–3 times/month	40 (11.4)	31 (15.6)	9 (5.9)	
Occasionally/never	4 (1.1)	4 (2.0)	0 (0.0)	
Processed				
1–6 times/day	73 (20.7)	40 (20.1)	33 (21.9)	.127
1–3 times/week	108 (30.7)	53 (26.6)	55 (36.4)	
1–3 times/month	143 (40.6)	88 (44.2)	55 (36.4)	
Occasionally/never	26 (7.4)	18 (9.0)	8 (5.3)	
Ultra‐processed				
1–6 times/day	104 (29.5)	52 (26.1)	52 (34.4)	**.043**
1–3 times/week	99 (28.1)	51 (25.6)	48 (31.8)	
1–3 times/month	112 (31.8)	71 (35.7)	41 (27.2)	
Occasionally/never	35 (9.9)	25 (12.6)	10 (6.6)	

*Note*: Bold values indicate p values ≤ 0.05.

Among male students, the frequency of consumption of unprocessed foods, ultra‐processed and high consumption of both unprocessed and ultra‐processed foods was significantly associated with blood pressure but this was not the case for female students. Among males with elevated blood pressure, 77.8% consumed unprocessed or minimally processed foods less frequently (1–3 times per month) and 76.9% consumed ultra‐processed foods daily. Among female students, the consumption of culinary ingredients was significantly associated with blood pressure. Among females who had normal blood pressure, 67.8% consumed culinary ingredients daily while 35.7% consumed them 1–3 times per week. For females with elevated blood pressure, 32.2% consumed culinary ingredients daily while 64.3% consumed culinary ingredients 1–3 times per month. NOVA food category consumption was not significantly associated with waist circumference and BMI status. Tables 3, 4, 5 show the association between NOVA food consumption frequency and metabolic outcomes.

**TABLE 3:** Association between consumption of NOVA food class and blood pressure.

NOVA categories and frequency of consumption	Male			Female		
	Blood pressure					
	Normal, *n* (%)	High, *n* (%)	*p*‐value	Normal, *n* (%)	High, *n* (%)	*p*‐value
Unprocessed/minimally processed						
1–6 Times/day	29 (37.7)	48 (62.3)	**.016**	40 (58.8)	28 (41.2)	.309
1–3 Times/week	41 (49.4)	42 (50.6)		34 (63.0)	20 (37.0)	
1–3 Times/month	8 (22.2)	28 (77.8)		18 (72.0)	7 (28.0)	
Rarely/Never	0 (0.0)	3 (100.0)		1 (25.0)	3 (75.0)	
Culinary ingredients						
1–6 Times/day	47 (38.8)	74 (61.2)	.704	78 (67.8)	37 (32.2)	**.007**
1–3 Times/week	19 (44.2)	24 (55.8)		10 (35.7)	18 (64.3)	
1–3 Times/month	10 (32.3)	21 (67.7)		6 (66.7)	3 (33.3)	
Rarely/Never	2 (50.0)	2 (50.0)		0 (0)	0 (0)	
Processed						
1–6 Times/day	12 (30.0)	28 (70.0)	.277	17 (51.5)	16 (48.5)	.349
1–3 Times/week	25 (47.2)	28 (52.8)		38 (69.1)	17 (30.9)	
1–3 Times/month	36 (40.9)	52 (59.1)		34 (61.8)	21 (38.2)	
Rarely/Never	5 (27.8)	13 (72.2)		4 (50.0)	4 (50.0)	
Ultra‐processed						
1–6 Times/day	12 (23.1)	40 (76.9)	**.020**	33 (63.5)	19 (36.5)	.505
1–3 Times/week	24 (47.1)	27 (52.9)		29 (60.4)	19 (39.6)	
1–3 Times/month	34 (47.9)	37 (52.1)		27 (65.9)	14 (34.1)	
Rarely/Never	8 (32.0)	17 (68.0)		4 (40.0)	6 (60.0)	
Ultra‐processed plus unprocessed						
High–high	35 (35.4)	64 (64.6)	**.001**	56 (62.2)	34 (37.8)	.758
High–low	36 (55.4)	29 (44.6)		24 (57.1)	18 (42.9)	
Low–low	7 (20.0)	28 (80.0)		12 (66.7)	6 (33.3)	

*Note*: Bold values indicate p values ≤ 0.05.

**TABLE 4:** Association between NOVA food class and waist circumference.

NOVA categories	Male			Female		
	Waist circumference					
	Normal, *n* (%)	Obese, *n* (%)	*p*‐value	Normal, *n* (%)	Obese *n* (%)	*p*‐value
Unprocessed/minimally processed						
1–6 Times/day	75 (97.4)	2 (2.6)	.683	61 (91.0)	6 (9.0)	.285
1–3 Times/week	81 (97.6)	2 (2.4)		43 (79.6)	11 (20.4)	
1–3 Times/month	34 (94.4)	2 (5.6)		22 (88.0)	3 (12.0)	
Rarely/Never	3 (100)	0 (0.0)		4 (100.0)	0 (0.0)	
Culinary ingredients						
1–6 Times/day	118 (97.5)	3 (2.5)	.106	103 (90.4)	11 (9.6)	.057
1–3 Times/week	41 (95.3)	2 (4.7)		21 (75)	7 (25)	
1–3 Times/month	31 (100)	0 (0.0)		7 (77.8)	2 (22.2)	
Rarely/Never	3 (75.0)	1 (25.0)		0 (0)	0 (0)	
Processed						
1–6 Times/day	39 (97.5)	1 (2.5)	.924	29 (87.9)	4 (12.1)	.880
1–3 Times/week	52 (98.1)	1 (1.9)		47 (85.5)	8 (14.5)	
1–3 Times/month	84 (95.5)	4 (4.5)		46 (85.2)	8 (14.8)	
Rarely/Never	18 (100.0)	0 (0.0)		8 (100.0)	0 (0.0)	
Ultra‐processed						
1–6 Times/day	51 (98.1)	1 (1.9)	.795	47 (90.4)	5 (9.6)	.578
1–3 Times/week	50 (98.0)	1 (2.0)		41 (87.2)	6 (12.8)	
1–3 Times/month	68 (95.8)	3 (4.2)		33 (80.5)	8 (19.5)	
Rarely/Never	24 (96.0)	1 (4.0)		9 (90.0)	1 (10.0)	
Ultra‐processed plus unprocessed						
High–high	97 (98.0)	2 (2.0)	.657	79 (88.8)	10 (11.2)	.467
High–low	63 (96.9)	2 (3.1)		34 (81.0)	8 (19.0)	
Low–low	33 (94.3)	2 (5.7)		16 (88.9)	2 (11.1)	

**TABLE 5:** NOVA food consumption and weight status of study participants.

NOVA categories	Male					Female				
	Underweight, *n* (%)	Normal, *n* (%)	Overweight, *n* (%)	Obese, *n* (%)	*p*‐value	Underweight, *n* (%)	Normal, *n* (%)	Overweight, *n* (%)	Obese, *n* (%)	*p*‐value
Unprocessed/minimally processed										
1–6 Times/day	5 (6.5)	61 (79.2)	9 (11.7)	2 (2.6)	.999	9 (13.2)	37 (54.4)	16 (23.5)	6 (8.8)	.368
1–3 Times/week	7 (8.4)	65 (78.3)	9 (10.8)	2 (2.4)		5 (9.30)	24 (44.4)	15 (27.8)	10 (18.5)	
1–3 Times/month	2 (5.6)	29 (80.6)	4 (11.1)	1 (2.8)		7 (28.0)	11 (44.0)	4 (16.0)	3 (12.0)	
Rarely/Never	0 (0.0)	3 (100.0)	3 (100.0)	0 (0.0)		0 (0.0)	2 (50.0)	2 (50.0)	0 (0.0)	
Culinary Ingredients										
1–6 Times/day	10 (8.3)	94 (77.7)	14 (11.6)	3(2.5)	.653	20 (17.4)	5 8(50.4)	25 (21.7)	12 (10.4)	.112
1–3 Times/week	3 (7.0)	36 (83.7)	2 (4.7)	2 (4.7)		2 (7.1)	10 (35.7)	9 (32.1)	7 (25.0)	
1–3 Times/month	1 (3.2)	24 (77.4)	6 (19.4)	0 (0.0)		0 (0.0)	6 (66.7)	3 (33.3)	0 (0.0)	
Rarely/Never	0 (0.0)	4 (100)	0 (0.0)	0 (0.0)		0 (0)	0 (0)			
Processed										
1–6 Times/day	1 (2.5)	35 (87.5)	4 (10.0)	0 (0.0)	.847	5 (15.2)	1 8(54.5)	6 (18.2)	4 (12.1)	.550
1–3 Times/week	5 (9.4)	42 (79.2)	5 (9.4)	1 (1.9)		5 (9.1)	31 (56.4)	12 (21.8)	7 (12.7)	
1–3 Times/month	6 (6.8)	67 (76.1)	11 (12.5)	4 (4.5)		10 (18.2)	20 (36.4)	17 (30.9)	8 (14.5)	
Rarely/Never	2 (11.1)	14 (77.8)	2 (11.1)	0 (0.0)		2 (25)	4 (50.0)	2 (25.0)	0 (0.0)	
Ultra‐processed										
1–6 Times/day	1 (1.9)	42 (80.8)	8 (15.4)	1 (1.9)	.248	10 (19.2)	29 (55.8)	8 (15.4)	5 (9.6)	.464
1–3 Times/week	5 (9.8)	38 (74.5)	7 (13.7)	1 (2.0)		3 (6.2)	23 (47.9)	15 (31.2)	7 (14.6)	
1–3 Times/month	4 (5.6)	60 (84.5)	4 (5.6)	3 (4.2)		7 (17.1)	17 (41.5)	1 (26.8)	6 (14.6)	
Rarely/Never	4 (16.0)	18 (72.0)	3 (12.0)	0 (0.0)		1 (10.0)	5 (50.0)	3 (30.0)	1 (10.0)	
Ultra‐processed plus unprocessed										
High–high	6 (6.1)	76 (76.8)	15 (15.2)	12 (13.3)	.374	12 (13.3)	47 (52.2)	20 (22.2)	11 (12.2)	.374
High–low	6 (9.2)	54 (83.1)	3 (4.6)	3 (7.1)		3 (7.1)	19 (45.2)	14 (33.3)	6 (14.3)	
Low–low	2 (5.7)	28 (80.0)	4 (11.4)	5 (27.8)		5 (27.8)	8 (44.4)	3 (16.7)	2 (11.1)	

Table 6 shows the meal consumption patterns and perceived barriers to healthy food consumption among students. The majority of study participants did not consume breakfast before leaving their hostel or home nor did they plan their meals in advance. These findings were similar among males and females. Also, the majority of the students indicated that the period within the day they had adequate time to eat was in the afternoon (54.3%) and evening (38.9%) compared to the morning (6.8%). More than half (52.7%) of the study participants indicated that fast foods constituted their favorite foods and this was followed by local/traditional foods (29.3%). For the barriers to healthy food consumption, a high proportion of participants strongly agreed with the statements healthy foods are more expensive (29%), cooking healthy foods is more difficult compared to cooking processed/ultra‐processed foods (28.1%), and limited time to prepare healthy foods (26.4%) and nonperiodical follow‐up on nutritional status (27.3%). The majority of students strongly disagreed with the statement succumbing to peer pressure (27.0%) and the statement university food environment is not suitable for consuming a healthy diet (31.3%).

**TABLE 6:** Meal consumption and perceived barriers to healthy food consumption.

Perceived barriers	Total *N* (%) *N* = 352 (100)	Male, *n* (%)	Female, *n* (%)	*p*‐value
Breakfast consumption before leaving home/hostel				
Yes	92 (26.2)	57 (28.6)	35 (23)	.271
No	259 (73.8)	142 (71.4)	117 (77.0)	
Meal planning in advance				
Yes	148 (42)	79 (39.7)	69 (45.1)	.328
No	204 (58)	120 (60.3)	84 (54.9)	
Period in the day there is adequate time to eat				
Morning	24 (6.8)	14 (7.0)	10 (6.5)	.316
Afternoon	191 (54.3)	101 (50.8)	90 (58.8)	
Evening	137 (38.9)	84 (42.2)	53 (34.6)	
What constitutes your favorite meal?				
Snacks	21 (6)	11 (5.5)	10 (6.6)	.987
Processed/ultra‐processed foods	42 (12)	24 (12.1)	18 (11.8)	
Local/traditional foods	103 (29.3)	59 (29.6)	44 (28.9)	
Fast foods	185 (52.7)	105 (52.8)	80 (52.6)	
Succumbing to peer pressure				
Strongly agree	31 (8.8)	15 (7.6)	16 (10.7)	**<.001**
Agree	54 (15.3)	26 (13.1)	28 (18.7)	
Neutral	75 (21.3)	61 (30.8)	14 (9.3)	
Disagree	93 (26.4)	45 (22.7)	48 (32.0)	
Strongly disagree	95 (27.0)	51 (25.8)	44 (29.3)	
Cooking healthy food is more difficult compared to cooking processed/ultra‐processed foods				
Strongly agree	99 (28.1)	56 (28.1)	43 (28.3)	.596
Agree	89 (25.3)	51 (25.6)	38 (25.00	
Neutral	50 (14.2)	33 (16.3)	17 (11.2)	
Disagree	55 (15.6)	28 (14.1)	27 (17.8)	
Strongly disagree	58 (16.5)	31 (15.6)	27 (17.8)	
Nonperiodical follow‐up of my nutritional status				
Strongly agree	96 (27.3)	51 (25.6)	45 (29.4)	.437
Agree	88 (25.0)	47 (23.6)	41 (26.8)	
Neutral	85 (24.1)	55 (27.6)	30 (19.6)	
Disagree	51 (14.5)	30 (15.1)	21 (13.7)	
Strongly disagree	32 (9.1)	16 (8.0)	16 (10.5)	
Unhealthy diet used as coping strategy for stress				
Strongly agree	59 (16.8)	25 (12.6)	34 (22.2)	**.036**
Agree	67 (19.0)	41 (20.6)	26 (17.0)	
Neutral	79 (22.4)	52 (26.1)	27 (17.6)	
Disagree	82 (23.3)	41 (20.6)	41 (26.8)	
Strongly disagree	65 (18.5)	40 (20.1)	25 (16.3)	
Limited time to prepare healthy food				
Strongly agree	93 (26.4)	46 (23.1)	47 (30.7)	.450
Agree	70 (19.9)	45 (22.6)	25 (16.3)	
Neutral	67 (19.0)	38 (19.1)	29 (19.0)	
Disagree	69 (19.6)	39 (19.6)	30 (19.6)	
Strongly disagree	53 (15.1)	31 (15.6)	22 (14.4)	
Healthy foods are expensive				
Strongly agree	102 (29.0)	54 (27.1)	48 (31.6)	.665
Agree	70 (19.9)	43 (21.6)	27 (17.8)	
Neutral	66 (18.8)	41 (20.6)	25 (16.4)	
Disagree	64 (18.2)	35 (17.6)	29 (19.1)	
Strongly disagree	49 (13.9)	26 (13.1)	23 (15.1)	
Inadequate knowledge/Information about healthy diet				
Strongly agree	57 (16.2)	35 (17.6)	22 (14.4)	**.013**
Agree	81 (23.0)	52 (26.1)	29 (19.0)	
Neutral	79 (22.4)	49 (24.6)	30 (19.6)	
Disagree	87 (24.7)	46 (23.1)	41 (26.8)	
Strongly disagree	48 (13.6)	17 (8.5)	31 s(20.3)	
University food environment not suitable for eating Healthy Diet				
Strongly agree	57 (16.2)	29 (14.6)	28 (18.3)	.461
Agree	43 (12.2)	26 (13.1)	17 (11.1)	
Neutral	61 (17.3)	39 (19.6)	22 (14.4)	
Disagree	81 (23.0)	48 (24.1)	33 (21.6)	
Strongly disagree	110 (31.3)	57 (28.6)	53 (34.6)	

*Note*: Bold values indicate p values ≤ 0.05.

### 3.1 Predictors of blood pressure

Table 7 shows the binary logistic regression of the predictors of blood pressure. Body mass index, gender, and consumption of unprocessed and ultra‐processed foods showed significant associations with blood pressure. Male gender was associated with significantly reduced odds for normal blood pressure compared to females (OR 0.190, CI 0.107–0.337, *p* < .001). High consumption of unprocessed foods was associated with reduced odds for normal blood pressure (OR 0.435, CI 0.220–0.861, *p* = .017).

**TABLE 7:** Binary logistic regression of the predictors of blood pressure.

	Explanatory variables	Odds ratio	*p*‐value
Model: Blood pressure+	Age	1.030 (0.927–1.144)	.588
	BMI	1.250 (1.158–1.350)	<.001
	Waist‐to‐hip ratio	0.908 (0.485–1.698)	.762
	Monthly income	1.000 (0.999–1.001)	.583
	*Gender*		
	Male	0.190 (0.107–0.337)	<.001
	Female^a^		
	*Unprocessed foods consumption*		
	High	0.435 (0.220–0.861)	.017
	Low^a^		
	*Ultra‐processed foods consumption*		
	High	1.926 (1.112–3.337)	.019
	Low^a^		

*Note*: Variable set as reference for blood pressure is elevated blood pressure.

^a^ Variable set as reference.

## 4 DISCUSSION

This study assessed the level of consumption of the four categories of NOVA food among students of the Takoradi Technical University located in the Western Region of Ghana and how they relate to weight and blood pressure as well as barriers to healthy food consumption faced by students. Findings from the study reveal that the prevalence of elevated pressure is high among the students while the prevalence of overweight and obesity is lower than what is reported among the general adult population (Asosega et al., 2023; Atuahene et al., 2017; Ofori‐Asenso et al., 2016; Owiredu et al., 2019; Solomon et al., 2017; Tuoyire et al., 2016). The mean BMI of study participants was within the normal range but blood pressure was slightly above normal with males recording a significantly higher value. Though the blood pressure measurement of participants in this study was taken once, elevated blood pressure observed among them is of grave concern. Elevated blood pressure among the students can be linked to a reliance on out‐of‐home cooked meals which tend to be high in salt and fat and or skipping breakfast which is linked to snacking and other inappropriate habits later in the day. Additionally, male students are more likely to depend on out‐of‐home cooked foods compared to females and have lower intakes of vegetables which may make them more prone to elevated blood pressure (Mello Rodrigues et al., 2019). Elevated blood pressure in adolescence is linked to high pulse, thickened carotid intima‐media, hypertrophy of left ventricular, and cardiovascular disease‐related mortality in adulthood (Yang et al., 2020). In a similar study among college students in Ethiopia, Tadesse and Alemu (2014) reported that the male gender was associated with a higher risk of hypertension. Similar findings have also been reported among university students in Congo where the prevalence of hypertension was 26.4% (Lebbie et al., 2017). In Ghana, hypertension awareness tends to be lower among younger persons compared to older adults probably resulting from their perceived lower risk of acquiring the condition (Sanuade et al., 2020). This calls for public health education and routine screening among adolescents and young adults to conscientize them on their blood pressure levels and the need to adopt healthful lifestyle. This is especially essential as majority of students strongly agreed that non‐periodical follow‐up on their nutritional status was a barrier to healthy food consumption. Hypertension is a risk factor for several chronic diseases including stroke, kidney diseases, and heart diseases, and one of the leading causes of death in Ghana (Owusu et al., 2021). The prevalence of overweight and obesity was significantly higher among females compared to males. Several studies conducted among adolescents and adults within and outside Ghana have reported a higher prevalence of overweight and obesity among the female gender compared to the male gender (Lartey et al., 2020; Ofori‐Asenso et al., 2016).

Furthermore, findings on consumption revealed that students had a higher frequency of consumption of unprocessed foods as well as ultra‐processed foods. This implies that participants consume protective foods such as vegetables, fruits, whole grains, and other whole foods but at the same time consume lots of unhealthy options such as sugar‐sweetened drinks and biscuits among others. The high consumption of ultra‐processed foods has the potential to negate the positive health impact of unprocessed food consumption and thus puts students at risk of adverse health outcomes. Ultra‐processed foods are high in fat, salt, and sugar and these increase the risk of hypertension, obesity, and other chronic diseases. A study conducted by Cordova et al. (2021) revealed that the consumption of ultra‐processed foods is strongly associated with a 5‐year increase in weight in a dose–response manner. In this study, the beneficial impact of unprocessed food consumption on health was not clearly evident. In fact, among males, the majority of those with a consumption frequency of 1–6 times per day for unprocessed foods had elevated blood pressure. Unprocessed foods are most effective in promoting cardiometabolic health when they are consumed frequently as part of an overall healthy dietary pattern and lifestyle such as regular physical activity (Smiljanec et al., 2020). High consumption of ultra‐processed food is linked to excessive caloric intake and inadequate consumption of essential nutrients (Cattafesta et al., 2020; Rossato et al., 2023). Additionally, among female participants, the frequency of consumption of culinary ingredients was higher among those with normal blood pressure. Culinary ingredients such as sugar may be added to whole‐grain porridges like oats, wheat, or corn. Vegetable oil and animal fat are often used in the preparation of gravies, most of which are vegetable based. Their health impacts are also dependent on the quantities consumed. It is, therefore, possible that the female participants with normal blood pressure and perhaps study participants in general did not consume high quantities of these foods. The majority of students did not consume breakfast nor did they plan their meals; these practices predisposed them to high consumption of ultra‐processed foods as most of them also indicated that fast foods constituted their favorite meal. Individuals who plan their meals tend to have healthier and varied diets as well as decreased odds for adverse metabolic outcomes (Ducrot et al., 2017).

Students strongly disagreed with the unsuitability of the university food environment and peer pressure as a barrier to healthy food consumption; however, they strongly agreed with healthy foods being more difficult to cook compared to junk foods, limited time to prepare healthy foods, and healthy foods being expensive. These findings presuppose that the university food environment is not perceived as a barrier to healthy food consumption but rather the prices of healthy foods in addition to limited time to cook these foods. The price of foods influences their selection and purchase. Affordability of ultra‐processed foods leads to their frequent purchase and consumption in spite of their negative impact on health. On the other hand, the high cost of unprocessed/minimally processed foods such as whole grains, vegetables, and fruits is linked to their low levels of purchase and consumption. Globally, healthy diets cost about five times more compared to unhealthy diets and their total cost is even higher than the international poverty line (Herforth et al., 2020). Establishments of university canteens and food outlets that provide healthy affordable foods to students, teaching students on time management skills and nutrition education can help to mitigate barriers to healthy food consumption (Deliens et al., 2014; McPherson et al., 2022).

## 5 CONCLUSION

The frequency of consumption of unprocessed foods was high among students but this was accompanied by high consumption of ultra‐processed foods. About 50% of students had elevated blood pressure with males recording a significant value. Among males, high consumption of ultra‐processed foods was associated with elevated blood pressure. The beneficial impact of unprocessed foods was not seen in this study and it is likely due to the high consumption of ultra‐processed foods along with unprocessed foods.

## AUTHOR CONTRIBUTIONS

**Elizabeth Sekyi:** Conceptualization (equal); data curation (equal); formal analysis (equal); investigation (equal); methodology (equal); resources (equal); validation (equal); visualization (equal); writing – original draft (equal); writing – review and editing (equal). **Nana Ama Frimpomaa Agyapong:** Conceptualization (equal); data curation (equal); formal analysis (equal); investigation (equal); methodology (equal); project administration (equal); software (equal); supervision (equal); validation (equal); visualization (equal); writing – original draft (equal); writing – review and editing (equal). **Guy Eshun:** Conceptualization (equal); data curation (equal); investigation (equal); methodology (equal); supervision (equal); writing – original draft (equal); writing – review and editing (equal).

## CONFLICT OF INTEREST STATEMENT

Elizabeth Sekyi is a lecturer at the Takoradi Technical University. However, the study was conducted objectively and the results obtained analyzed by all contributing authors to ensure that results are not manipulated or falsified. The two other co‐authors declare no conflicting interest.

## Data Availability

The data for the study can be made available by the corresponding author upon request.

## References

[fsn33894-bib-0001] Abdelhafez, A. I. , Akhter, F. , Alsultan, A. A. , Jalal, S. M. , & Ali, A. (2020). Dietary practices and barriers to adherence to healthy eating among King Faisal University students. International Journal of Environmental Research and Public Health, 17(23), 8945. 33271893 10.3390/ijerph17238945 PMC7731134

[fsn33894-bib-0002] Asosega, K. A. , Aidoo, E. N. , Adebanji, A. O. , & Owusu‐Dabo, E. (2023). Examining the risk factors for overweight and obesity among women in Ghana: A multilevel perspective. Heliyon, 9(5), e16207. 37229171 10.1016/j.heliyon.2023.e16207 PMC10205511

[fsn33894-bib-0003] Atuahene, M. , Ganle, J. K. , Adjuik, M. , Atuahene, N. F. , & Kampitib, G. B. (2017). Overweight and obesity prevalence among public servants in Nadowli district, Ghana, and associated risk factors: A cross‐sectional study. BMC Obesity, 4(1), 1–8. 28572982 10.1186/s40608-017-0153-5 PMC5452290

[fsn33894-bib-0004] Baker, P. , Machado, P. , Santos, T. , Sievert, K. , Backholer, K. , Hadjikakou, M. , Russell, C. , Huse, O. , Bell, C. , Scrinis, G. , Worsley, A. , Friel, S. , & Lawrence, M. (2020). Ultra‐processed foods and the nutrition transition: Global, regional and national trends, food systems transformations and political economy drivers. Obesity Reviews, 21(12), e13126. 32761763 10.1111/obr.13126

[fsn33894-bib-0005] Cattafesta, M. , Petarli, G. B. , Zandonade, E. , Bezerra, O. M. D. P. A. , Abreu, S. M. R. D. , & Salaroli, L. B. (2020). Energy contribution of NOVA food groups and the nutritional profile of the Brazilian rural workers' diets. PLoS One, 15(10), e0240756. 33112887 10.1371/journal.pone.0240756 PMC7592810

[fsn33894-bib-0006] Chen, X. , Zhang, Z. , Yang, H. , Qiu, P. , Wang, H. , Wang, F. , Zhao, Q. , Fang, J. , & Nie, J. (2020). Consumption of ultra‐processed foods and health outcomes: A systematic review of epidemiological studies. Nutrition Journal, 19(1), 1–10. 32819372 10.1186/s12937-020-00604-1 PMC7441617

[fsn33894-bib-0007] Chuan, C. L. , & Penyelidikan, J. (2006). Sample size estimation using Krejcie and Morgan and Cohen statistical power analysis: A comparison. Jurnal Penyelidikan IPBL, 7(1), 78–86.

[fsn33894-bib-0008] Cordova, R. , Kliemann, N. , Huybrechts, I. , Rauber, F. , Vamos, E. P. , Levy, R. B. , Wagner, K. H. , Viallon, V. , Casagrande, C. , Nicolas, G. , Dahm, C. C. , Zhang, J. , Halkjær, J. , Tjønneland, A. , Boutron‐Ruault, M. C. , Mancini, F. R. , Laouali, N. , Katzke, V. , Srour, B. , … Freisling, H. (2021). Consumption of ultra‐processed foods associated with weight gain and obesity in adults: A multi‐national cohort study. Clinical Nutrition, 40(9), 5079–5088. 34455267 10.1016/j.clnu.2021.08.009

[fsn33894-bib-0009] Deliens, T. , Clarys, P. , De Bourdeaudhuij, I. , & Deforche, B. (2014). Determinants of eating behaviour in university students: A qualitative study using focus group discussions. BMC Public Health, 14(1), 1–12. 24438555 10.1186/1471-2458-14-53 PMC3905922

[fsn33894-bib-0010] Ducrot, P. , Méjean, C. , Aroumougame, V. , Ibanez, G. , Allès, B. , Kesse‐Guyot, E. , Hercberg, S. , & Péneau, S. (2017). Meal planning is associated with food variety, diet quality and body weight status in a large sample of French adults. International Journal of Behavioral Nutrition and Physical Activity, 14(1), 1–12. 28153017 10.1186/s12966-017-0461-7 PMC5288891

[fsn33894-bib-0011] Food and Agriculture Organization of the United Nations . (2016). UN General Assembly proclaims Decade of Action on Nutrition . http://www.fao.org/news/story/en/item/408970/icode/

[fsn33894-bib-0013] Herforth, A. , Bai, Y. , Venkat, A. , Mahrt, K. , Ebel, A. , & Masters, W. A. (2020). Cost and affordability of healthy diets across and within countries: Background paper for the state of food security and nutrition in the world 2020 . FAO agricultural development economics technical study No. 9 (Vol. 9). Food & Agriculture Org.

[fsn33894-bib-0014] Isa, K. A. M. , & Masuri, M. G. (2011). The association of breakfast consumption habit, snacking behavior and body mass index among university students. American Journal of Food and Nutrition, 1(2), 55–60.

[fsn33894-bib-0015] Lartey, S. , Si, L. , Lung, T. , Magnussen, C. G. , Boateng, G. O. , Minicuci, N. , Kowal, P. , Hayes, A. , de Graaff, B. , Blizzard, L. , & Palmer, A. J. (2020). Impact of overweight and obesity on life expectancy, quality‐adjusted life years and lifetime costs in the adult population of Ghana. BMJ Global Health, 5(9), e003332. 10.1136/bmjgh-2020-003332 PMC7526271 32994229

[fsn33894-bib-0016] Lebbie, A. , Wadsworth, R. , Saidu, J. , & Bangura, C. (2017). Predictors of hypertension in a population of undergraduate students in Sierra Leone. International Journal of Hypertension, 2017, 8196362. 28840040 10.1155/2017/8196362 PMC5559978

[fsn33894-bib-0017] Lima, J. P. , Costa, S. A. , Brandão, T. R. , & Rocha, A. (2021). Food consumption determinants and barriers for healthy eating at the workplace—A university setting. Food, 10(4), 695. 10.3390/foods10040695 PMC8064356 33805929

[fsn33894-bib-0018] McPherson, M. , Lai, C. C. , Leahy, D. , Wessell, A. , Srinivasan, S. , Hanley, B. , Peeters, A. , & Palermo, C. (2022). Facilitators to healthy canteen policy implementation: A qualitative approach. Health Promotion Journal of Australia, 33(1), 216–223. 33561895 10.1002/hpja.465

[fsn33894-bib-0019] Mello Rodrigues, V. , Bray, J. , Fernandes, A. C. , Luci Bernardo, G. , Hartwell, H. , Secchi Martinelli, S. , Lazzarin Uggioni, P. , Barletto Cavalli, S. , & Proença, R. P. D. C. (2019). Vegetable consumption and factors associated with increased intake among college students: A scoping review of the last 10 years. Nutrients, 11(7), 1634. 31319573 10.3390/nu11071634 PMC6682864

[fsn33894-bib-0020] Menegassi, B. , de Morais Sato, P. , Scagliusi, F. B. , & Moubarac, J. C. (2019). Comparing the ways a sample of Brazilian adults classify food with the NOVA food classification: An exploratory insight. Appetite, 137, 226–235. 30862456 10.1016/j.appet.2019.03.010

[fsn33894-bib-0021] Momanyi, M. M. , & Akinyi, S. A. (2014). Influence of dietary patterns on nutritional status among female students in Maseno Univerity . https://www.academia.edu/8283139/INFLUENCE_OF_DIETARY_PATTERNS_ON_NUTRITIONAL_STATUS_AMONG_FEMALE_STUDENTS_IN_MASENO_UNIVERITY

[fsn33894-bib-0022] Monteiro, C. A. , Cannon, G. , Moubarac, J. C. , Levy, R. B. , Louzada, M. L. C. , & Jaime, P. C. (2018). The UN decade of nutrition, the NOVA food classification and the trouble with ultra‐processing. Public Health Nutrition, 21(1), 5–17. 28322183 10.1017/S1368980017000234 PMC10261019

[fsn33894-bib-0023] Musaiger, A. O. , Al‐Mannai, M. , Tayyem, R. , Al‐Lalla, O. , Ali, E. Y. , Kalam, F. , Benhamed, M. M. , Saghir, S. , Halahleh, I. , Djoudi, Z. , & Chirane, M. (2013). Perceived barriers to healthy eating and physical activity among adolescents in seven Arab countries: A cross‐cultural study. The Scientific World Journal, 2013, 232164. 24348144 10.1155/2013/232164 PMC3848306

[fsn33894-bib-0024] Ofori‐Asenso, R. , Agyeman, A. A. , Laar, A. , & Boateng, D. (2016). Overweight and obesity epidemic in Ghana—A systematic review and meta‐analysis. BMC Public Health, 16(1), 1–18. 27938360 10.1186/s12889-016-3901-4 PMC5148846

[fsn33894-bib-0025] Owiredu, E. W. , Dontoh, E. , Essuman, S. E. , & Bazanfara, B. B. (2019). Demographic and lifestyle predictors of prehypertension: A cross‐sectional study among apparently healthy adults in Kumasi, Ghana. BioMed Research International, 2019, 1764079. 31179316 10.1155/2019/1764079 PMC6507075

[fsn33894-bib-0026] Owusu, A. Y. , Kushitor, S. B. , Ofosu, A. A. , Kushitor, M. K. , Ayi, A. , & Awoonor‐Williams, J. K. (2021). Institutional mortality rate and cause of death at health facilities in Ghana between 2014 and 2018. PLoS One, 16(9), e0256515. 34496000 10.1371/journal.pone.0256515 PMC8425528

[fsn33894-bib-0027] Pagliai, G. , Dinu, M. , Madarena, M. P. , Bonaccio, M. , Iacoviello, L. , & Sofi, F. (2021). Consumption of ultra‐processed foods and health status: A systematic review and meta‐analysis. British Journal of Nutrition, 125(3), 308–318. 32792031 10.1017/S0007114520002688 PMC7844609

[fsn33894-bib-0028] Rossato, S. L. , Khandpur, N. , Lo, C. H. , Castro, S. M. J. , Drouin‐Chartier, J. P. , Sampson, L. , Yuan, C. , Murta‐Nascimento, C. , Carvalhaes, M. A. , Monteiro, C. A. , Sun, Q. , Fung, T. T. , & Willett, W. C. (2023). Intakes of unprocessed and minimally processed and Ultraprocessed food are associated with diet quality in female and male health professionals in the United States: A prospective analysis. Journal of the Academy of Nutrition and Dietetics, 123, 1140.e2–1151.e2. 36965524 10.1016/j.jand.2023.03.011

[fsn33894-bib-0029] Roth, G. A. , Abate, D. , Abate, K. H. , Abay, S. M. , Abbafati, C. , Abbasi, N. , Abbastabar, H. , Abd‐Allah, F. , Abdela, J. , Abdelalim, A. , & Borschmann, R. (2018). Global, regional, and national age‐sex‐specific mortality for 282 causes of death in 195 countries and territories, 1980–2017: a systematic analysis for the Global Burden of Disease Study 2017. The Lancet, 392(10159), 1736–1788. 10.1016/S0140-6736(18)32203-7 PMC6227606 30496103

[fsn33894-bib-0030] Sanuade, O. A. , Awuah, R. B. , & Kushitor, M. (2020). Hypertension awareness, treatment and control in Ghana: A cross‐sectional study. Ethnicity & Health, 25(5), 702–716. 29448808 10.1080/13557858.2018.1439898

[fsn33894-bib-0031] Smiljanec, K. , Mbakwe, A. U. , Ramos‐Gonzalez, M. , Mesbah, C. , & Lennon, S. L. (2020). Associations of ultra‐processed and unprocessed/minimally processed food consumption with peripheral and central hemodynamics and arterial stiffness in young healthy adults. Nutrients, 12(11), 3229. 33105677 10.3390/nu12113229 PMC7690393

[fsn33894-bib-0032] Solomon, I. , Adjuik, M. , Takramah, W. , Axame, W. K. , Owusu, R. , Parbey, P. A. , Takase, M. , Tarkang, E. E. , & Kweku, M. (2017). The Frequency of Hypertension and Pre‐Hypertension among Adults in the Hohoe Municipality of Ghana . https://ir.ucc.edu.gh/xmlui/bitstream/handle/123456789/5477/The%20Frequency%20of%20Hypertension%20and%20Pre‐hypertension%20Among%20Adults%20in%20the%20Hohoe%20Municipality%20of%20Ghana.pdf?sequence=1&isAllowed=y

[fsn33894-bib-0033] Sprake, E. F. , Russell, J. M. , Cecil, J. E. , Cooper, R. J. , Grabowski, P. , Pourshahidi, L. K. , & Barker, M. E. (2018). Dietary patterns of university students in the UK: A cross‐sectional study. Nutrition Journal, 17(1), 1–17. 30290816 10.1186/s12937-018-0398-y PMC6172790

[fsn33894-bib-0034] Tadesse, T. , & Alemu, H. (2014). Hypertension and associated factors among university students in Gondar, Ethiopia: A cross‐sectional study. BMC Public Health, 14(1), 1–5. 25201163 10.1186/1471-2458-14-937 PMC4168247

[fsn33894-bib-0035] Tuoyire, D. A. , Kumi‐Kyereme, A. , & Doku, D. T. (2016). Socio‐demographic trends in overweight and obesity among parous and nulliparous women in Ghana. BMC Obesity, 3(1), 1–14. 27826451 10.1186/s40608-016-0124-2 PMC5093993

[fsn33894-bib-0037] World Health Organization . (2010). A healthy lifestyle ‐ WHO recommendations . https://www.who.int/europe/news‐room/fact‐sheets/item/a‐healthy‐lifestyle‐‐‐who‐recommendations

[fsn33894-bib-0038] World Health Organization . (2023). Hypertension . https://www.who.int/news‐room/fact‐sheets/detail/hypertension

[fsn33894-bib-0039] Yang, L. , Magnussen, C. G. , Yang, L. , Bovet, P. , & Xi, B. (2020). Elevated blood pressure in childhood or adolescence and cardiovascular outcomes in adulthood: A systematic review. Hypertension, 75(4), 948–955. 32114851 10.1161/HYPERTENSIONAHA.119.14168

